# Email Overload? Brain and Behavioral Responses to Common Messaging Alerts Are Heightened for Email Alerts and Are Associated With Job Involvement

**DOI:** 10.3389/fpsyg.2018.01206

**Published:** 2018-07-31

**Authors:** Maria Uther, Michelle Cleveland, Rhiannon Jones

**Affiliations:** Department of Psychology, University of Winchester, Winchester, United Kingdom

**Keywords:** work stress, attention, auditory alerts, messaging alerts, MMN, P3a

## Abstract

We tested brain and behavioral responses to two common messaging alerts (Outlook and Android whistle) using an oddball paradigm, where participants had to detect the two alerts among a background of white noise and occasional matched, distractor stimuli. Twenty-nine participants were tested using a behavioral target detection task and a subset of 14 were tested both with event-related potential (ERP) and behavioral oddball detection. For the ERP recordings, participants were instructed to attend to a distractor DVD in one condition and in the other, to actively attend to the stimuli. We measured mismatch negativity (MMN) and P3a components and questionnaire responses to job involvement, rumination and work-life balance. There were significantly larger MMN responses to target alert signals, but only in the ignore condition. In both ignore and attend conditions, MMN was larger for the Android stimuli, probably as a result of the larger physical discriminability for the Android tone. On the other hand, there was a significant P3a for Outlook tones, but not for Android tones in the ignore condition. Neither alert showed significant P3a activity within the attend condition, but instead later frontal positivity, which was larger for the Outlook alert (in comparison to its matched distractor) and this effect was not seen for the Android tones. This was despite the Outlook alert being less perceptually discriminable compared to the Android alert. These findings suggest that the indices of attentional processing are more affected by the significance of the alert than the physical qualities. These effects were coupled with the finding that the faster reaction times to the Outlook sounds were correlated with greater job involvement. These data suggest that work-related messages might signal greater attentional switch and effort which in turn may feed into greater job involvement.

## Introduction

The use of new communication technologies (NCTs) is an increasingly important part of our personal and work lives. An important part of the use of these technologies is the attention we give to alerts of new messages. NCTs often involve some kind of alert or notification (usually auditory, often coupled with visual, unless the user elects to turn them off). Although several researchers have studied the stress from the use of these technologies (e.g., Mazmanian et al., 2006; Park and Jex, 2011; Park et al., 2011; Stawarz et al., 2013; Collins et al., 2015), the focus has been on overall, longer term effects. There is by contrast very little research on the immediate nature of the disruption of these alerts.

A study of the immediate effect of messaging notifications at work and at home which would potentially account for how a barrage of messaging communication might potentiate longer term stress. There already exists two relevant theories [border and boundary theories see Ashforth et al. (2000), Clark (2000)], that account for long term ‘crossing’ of work and home lives and a sense that work permeates home life (often unhelpfully so- see Park and Jex, 2011; Park et al., 2011). Moreover, this work would also answer the question of whether reducing immediate work-related communication strain reduces longer term stress and a sense of ‘boundary keeping’ (Park and Jex, 2011; Park et al., 2011). An investigation of the neural response to these alerts is critical and timely, as it is possible that if indices of hypervigilance were found, that these might culminate in long term stress, contributing to wellbeing and productivity issues. If links were found between cognitive processing of such alerts and long term indices of job involvement/wellbeing, this may provide initial evidence for the hypothesis that excessive short term arousal/readiness is linked to longer term wellbeing at work issues.

One way of investigating the potential immediate disruption caused by NCT alerts is the use of event-related potentials (ERPs). ERPs allow the recording of neural activity in response to events (e.g., presentation of a certain stimulus or kind of stimulus) (Luck, 2014). The advantage of using ERPs is that the neural responses can be elicited in the absence of conscious, directed attention to stimuli. Of particular interest to studying auditory alerts are two types of event-related potential responses: the mismatch negativity (MMN) and the P3 components.

The MMN component of the auditory event-related potential is elicited in response to a violation of expectancy within a standard stimulus context. Typically, MMN responses are elicited within an ‘oddball’ paradigm, whereby an infrequent ‘deviant’ stimulus is presented that physically (e.g., in stimulus duration, intensity or frequency) differs from other frequent ‘standard tones’ (e.g., a 500 Hz tone among a sequence of repetitive 1000 Hz tones). However, the property of ‘deviance’ is not constrained to physically different stimuli. Deviant stimuli can also differ from standard stimuli in a more abstract sense [e.g., a reversal of a descending sequence of tones into an ascending sequence of tones (for a review, see Garrido et al., 2009; Näätänen et al., 2011)]. The MMN component typically peaks around 200 ms and has a fronto-central distribution.

The traditional interpretation of the presence of an MMN response is that it signifies the development of a sensory ‘memory trace’ (Näätänen, 1992). This view has been later refined into a ‘model adjustment’ hypothesis (Winkler, 2007) whereby MMN is thought to be reflective of error detection, formed from a comparison between incoming auditory input and the memory trace of the prior sensory context. Another theory that accounts for MMN responses suggests that it represents activity from an ‘adaptation’ system, that is formed from afferent neuronal activity [i.e., MMN is considered to represent refractory effects from the earlier, N1 component being attenuated through habituation (May and Tiitinen, 2010)]. However, whilst explaining some phenomena, the adaptation hypothesis cannot alone explain all empirical findings related to MMN (see Garrido et al., 2009).

Recent work (Garrido et al., 2009) has posited a ‘predictive coding’ (see Friston, 2005; Garrido et al., 2009; Wacongne et al., 2011, 2012; Bendixen et al., 2012) framework that reconciles these two accounts of MMN. Under a predictive coding interpretation, a model is formed of the regularities within the auditory context which results in predictions of future events (Friston, 2005; Winkler, 2007; Garrido et al., 2009). According to the predictive coding account, it encompasses both adaptation and model-adjustment views. It is a hierarchical account that predicts that the generation of a top-down model of incoming stimuli (similar to model-adjustment hypothesis), which is coupled with a bottom-up monitoring of changes in synaptic connections at a lower level (similar to the adaptation hypothesis), see Garrido et al. (2009) for a review.

Another key property of the MMN response is that it is elicited in the absence of conscious and focused attention to incoming auditory stimuli (Näätänen et al., 2011). Hence, it is suggested that it reflects pre-attentive mechanisms that trigger attentional switches to particularly novel or surprising stimuli (Näätänen et al., 2011). This view is compatible with empirical findings suggesting that the P3a (or novelty-P3) response, is elicited when there is an involuntary attention shift to the stimulus deviance in MMN paradigms. Such P3a responses are elicited when the stimuli are particularly surprising or novel (Escera and Corral, 2007; Polich, 2007). The P3a is distinguished from the P3b response which is elicited in actively attended conditions where responses are required to targets.

Taken together, a study of the MMN and P3 (a/b) responses will be a valuable investigation of the extent to which NCT auditory alerts might be processed differently compared to other sounds, or even among themselves. It is important to note that the MMN whilst elicited in response to physical or abstract deviance with increased physical deviation yielding larger responses (see Garrido et al., 2009; Näätänen et al., 2011), the MMN can also be elicited in relation to the meaning or significance of a sound. A clear example of this is the MMN elicited in response to non-native phonemes (Naatanen et al., 1997; Cheour et al., 1998; Winkler et al., 1999; Ylinen et al., 2010). In the studies of MMN in relation to phonemes, the same physical deviance can elicit smaller or larger responses depending on whether that physical change is phonemically relevant in one’s own native language. Moreover, the MMN responses to non-native phonemes can be enhanced with phonetic training, e.g., (Ylinen et al., 2010). There are also other examples of MMN elicited to meaningful stimuli – e.g., wolf whistles (Frangos et al., 2005) or musical stimuli (Brattico et al., 2006; Tervaniemi et al., 2014). Together, these results suggest that MMN enhancement may occur either for larger physical changes or changes that signal a particularly meaningful or important acoustical signal.

Highly relevant to this study is the work of Roye et al. (2007, 2010, 2013), which has centered mainly on investigating mobile phone lerts. In an initial study, Roye et al. (2007) used a yoked design, whereby participants were presented with their own usual ringtone (personally significant sound) and a ringtone from another participant (non-significant sound). They found that in addition to the expected mismatch negativity (MMN) and P3a component (for which there were no differences between personally significant and non-significant sounds), there was an additional parietal positivity following the MMN only for the personally significant deviant stimuli. Within this study, participants did not directly attend to the stimuli but had their attention diverted toward a distractor, sub-titled DVD film.

In a follow up study, Roye et al. (2010) used a similar (yoked) design to the earlier 2007 study but used both ignore and attend conditions. Instead of focusing on MMN and positive components, they instead examined evoked gamma-band responses (EGBRs) and showed that there was enhanced evoked oscillatory activity in the 35–75 Hz band for one’s own personal ringtone. A more recent study (Roye et al., 2013) investigated the effect of training a new personally significant sound over a period of 1 month. They found in this study that participants ERP responses were differentiated between personally significant and non-significant sounds from about 200 ms after stimulus onset, even when the sounds were task irrelevant. Roye et al. (2013) distinguishes between the concept of ‘familiar’ and ‘personally significant’ sounds. ‘Significance,’ Roye argues, encompasses qualitative components, namely emotional and behavioral relevance. The authors acknowledge that the concept of significance is a rather broad term and not necessarily personal. For example, the sound of someone screaming may be considered universally significant. On the other hand, sounds may be personally significant in a subjective sense (e.g., a child or family member calling you).

This study sought to extend the previous work of Roye and colleagues and not only look at ERP responses to messaging sounds, but also to look at the potential correlation between cognitive processing (indexed by reaction time and *d*′) and measures of balancing work and family/personal life, job involvement, psychological detachment and rumination. Reaction time and *d*′ were chosen as behavioral indices that provide independent information about the processing of these alerts. Reaction time indexes the ‘readiness’ to respond to stimuli whereas *d*′ measures the perceptual (physical discrimination) sensitivity. Both measures of cognitive processing were considered important to measure, as it is entirely possible that responses to different stimuli can be responded to equally quick by participants and yet at the same time, the responses themselves more prone to error (*d*′ takes into account hit rate and false alarm rates). Two alerts were chosen as at the time of testing, they were popular alerts in daily use: one was from a mobile device (Android whistle) and another from the Microsoft Outlook email program (commonly found on PCs as well as mobile devices). We also included a matched distractor for each alert (sounds matched for complexity, but different to the original sound and therefore differed in meaning), so that we could separate the effects of meaning from physical discrimination effects.

The following hypotheses were suggested:

(1) That the ERP indices of pre-attentive processes (indexed by MMN) may differ between the two alerts as a function of significance or difference in perceptual discriminability between the two stimuli. If purely physical effects drove the MMN response, one would see equivalent MMN amplitudes to the target sounds vs. matched distractors. On the other hand, if meaning drove the MMN responses, one would see larger MMNs to targets compared to their matched distractors.
(2) That the ERP indices of attentional processing – i.e., P3a or possibly later positive components, as in Roye et al. (2007), would show greater response amplitude for NCT alerts compared to matched distractors.
(3) That there would be a positive correlation between high tendencies of problematic work-patterns/job involvement and rumination (as indexed by our questionnaires) and behavioral target detection performance.
(4) There would be a dissociation between RT and *d*′ responses, such that target/meaningful stimuli may have quicker reaction times despite a smaller *d*′.

## Materials and Methods

### Participants

Twenty-nine participants (6 male, 23 female) were recruited via a University of Winchester staff intranet advertisement and participants were given a £10 Amazon voucher as an incentive to participate. Fourteen participants (12 female, 2 male) participated in the EEG study and all 29 participants participated in the behavioral detection task with no EEG. All participants completed a questionnaire of device ownership, device use and the psychological measures of work and non-working life. Written informed consent was given and ethical approval from the University’s Ethics Committee was given prior to the commencement of the study. The participants had a right to withdraw at any time without penalty.

### Stimuli and Materials

The two ‘alert’ sounds were the Microsoft ‘Outlook’ sounds for windows and the Android ‘whistle’ sound. The Outlook sound was 1.2 s in duration and the Android whistle approximately 1.3 s in duration. To derive matched distractors, the original sounds were sine-vocoded (Souza and Rosen, 2009) using locally developed Matlab software. Each sound was digitally filtered into eight bands, using sixth-order Butterworth IIR filters spaced at equal basilar membrane distance (Greenwood, 1990) across a frequency range of 0.05–10 kHz. The output of each band was full-wave rectified and low-pass filtered backward–forward at 300 Hz to extract the amplitude envelope. Each envelope was then multiplied by a sine-wave carrier at the band center frequency of the original analysis filter. The resulting signal (envelope × carrier) was filtered using the same bandpass filter as for the first filtering stage. The RMS level was adjusted at the output of the filter to match the level of the original band-pass filtered speech. Finally, all eight individual signals were summed across bands. All sounds were normalized in duration and played at 65 dB SPL.

Participants completed a questionnaire, which comprised of four main sections. The first asked general demographics, such as; age and occupation, the second included psychological measures of; balancing work and family/personal life, rumination, job involvement and psychological detachment. The third required participants to rate their familiarity with common alert tones for devices and the final section asked participants about device use and ownership.

#### Balancing Work and Family/Personal Life

Satisfaction of the balance between work and home life was measured on a 5-point likert scale, ranging from 1 – very dissatisfied, to 5 – very satisfied; developed by Valcour (2007). Participants rated their overall satisfaction with five items, including: “the way you divide your time between work and personal or family life” and “the opportunity you have to perform your job well and yet be able to perform home-related duties adequately.” The final item, relating to opportunities to perform both areas of duty adequately, was originally developed by Rothausen (1994).

#### Rumination

The extent to which participants felt they ruminated about work outside of their working hours was measured using a subscale of the Irritation Scale (see, for example; Mohr et al., 2006). The rumination sub-scale is termed cognitive irritation by Mohr et al. (2006) and was measured via three questions, for example: “even at home I often think of my problems at work.” Participants rated these items on a 7-point likert scale, ranging from 1 – strongly disagree to 7 – strongly agree.

#### Job Involvement

Job involvement was assessed using the scale developed by Kanungo (1982). Participants responded using a six-point likert scale to 10 questions, including: “the most important things that happen to me involve my present job” and “to me, my job is only a small part of who I am.” Participants rated these items on a six-point likert scale, ranging from 1 – disagree to 6 – agree.

#### Psychological Detachment

The extent to which participants felt they could psychologically detach from work was measured using four items, which were developed as part of the Recovery Experience Questionnaire (Sonnentag and Kruel, 2006; Sonnentag and Fritz, 2007). Participants rated questions, such as: “outside of work hours I completely forget about work” and “outside of work hours I get a break from the demands of work,” on a 5-point likert scale, ranging from 1 – I do not agree at all, to 5 – I fully agree.

#### Sound Familiarity

Participants rated their familiarity with three common email/message alert sounds. Participants were asked to rate, on a 4-point likert scale, “how familiar are you with this sound?” (from 1 – not at all familiar, to 4 – extremely familiar) and on a dichotomous yes/no scale, “is this sound one of your primary email/message alerts?”.

#### Device Use and Ownership

To collate information on device ownership, participants listed all the devices they currently owned and the software running on each device. Device use was measured through 15 questions, developed for the purposes of this study. The questions were pertaining to whether a mobile phone, a desktop computer, or a portable computer device were used for the following: leisure purposes, work purposes, to receive work-related emails, or to send work-related emails (a sample question was: “how often do you use a mobile phone for leisure purposes?”), measured on a 5-point scale from 1 – never, to 4 – several times a day, or 5 – n/a; whether the sounds and alerts were turned on (a sample question was: do you have sounds and alerts activated on your mobile phone?”, measured on a 4-point likert scale from 1 – never, to 3 – always, or 4 – n/a; and whether the work-related functions were ever switched off [a sample question was: “if you use your mobile phone for work, do you ever turn the work-related functions (e.g., email alerts) off?”], measured on a 5-point likert scale from 1 – never, to 4 – always, or 5 – n/a.

### Procedure

#### EEG Study

Participants first completed the questionnaire of device ownership, device use and the psychological measures of work and non-working life, rumination and job involvement before the EEG recording. For the EEG recording, stimuli were presented in an oddball paradigm, with an ignore condition where the participants’ attention was directed at watching a sub-titled DVD documentary (with corresponding questionnaire following to ensure that they were focusing on the DVD content). This was followed by an attend condition where the participants were required to actively attend to the auditory stimuli being played and press a button in response to the defined target (either Outlook tone or Android Whistle tone). Within each condition (ignore and attend), the oddball sequence comprised of two blocks of 60 trials of target (NCT alert) stimuli and 60 matched distractor deviants, as well as 360 white noise bursts matched in duration and normalized with deviants for overall intensity. In this way, the target and matched distractor stimuli had 12.5% probability each. The stimuli were blocked, such that within each condition, the Outlook tones (and corresponding matched distractors) were presented in separate blocks to the Android Whistle (and corresponding matched distractors). This was done because there were a small difference (order of about 100 ms) between both stimuli (which were in turn approximately 1 s long) as we wished standards, matched distractors and corresponding target deviants to be matched exactly in duration to avoid duration change responses. The order of Outlook and Android Whistle tone blocks was counterbalanced across participants.

Stimuli were delivered using Sony Headphones in a quiet room. EEG was collected using BioSemi 64-channel system with a 256-Hz sampling rate and a 0.16–100 Hz filter. Eye movements monitored with bipolar EOG from the outer canthi of both eyes as well as below and above the left eye.

#### Behavioral Study

The procedure for the behavioral study was identical to the EEG study in terms of stimuli used and the oddball paradigm probabilities. If ERPs were collected, the behavioral responses were analyzed from the attend ERP condition. For participants undergoing EEG, the attend condition always followed the ignore condition (so as not to contaminate the ignore condition by drawing participants’ attention to the stimuli and therefore inadvertently enhancing amplitudes in the ignore condition), in accordance with standard experimental procedure for recording MMN. For those only participating in the behavioral data collection, they were only given an attend condition (responding to the messaging alert target) and did not have EEG recorded at all. The participants’ reaction times, hit rate and *d*′ were analyzed with respect to each target and corresponding distractors.

#### EEG Analysis

EEG was filtered offline with 1.0–30 Hz band pass filter (24 dB/octave) and re-referenced to the earlobes. ERPs were averaged using epochs using -100 to +500 ms stimulus onset. ERPs were baseline corrected -100 ms to 0 ms from stimulus onset. Epochs corresponding to behavioral errors in the Attending condition were rejected (misses for target trials; false alarms for non-target trials) and ICA was run to correct eye-movements and any remaining epochs containing artifacts were rejected semi-automatically with a ±70 μV threshold.

The MMN component was measured from difference waveforms (i.e., subtracting standard stimulus response from the deviant response). The MMN amplitudes for each individual were calculated over a mean of 40 ms epoch centered around the maximum amplitude in the region of about 150–250 ms in the grand average waveform for that particular condition (Ignore Outlook latency window: 128–168 ms; Ignore Android latency window: 199–239 ms; Attending Outlook latency window: 230–270 ms; Attending Android latency window: 195–235 ms). The P3 amplitudes were also measured from difference waveforms (i.e., subtracting standard stimulus response from the deviant response). The P3 amplitudes for each individual were measured from to the rarer deviant tone conditions and individual mean amplitudes were calculated over a 40 ms epoch centered around the maximum amplitude in the region of about 230–350 ms in the grand average waveform for that condition (Ignore Outlook latency window: 292–332 ms; Ignore Android latency window: 292–332 ms; Attending Outlook latency window: 304–344 ms; Attending Android latency window: 331–377 ms). A late positive component was found in some conditions at 398 ms and mean amplitudes were also calculated in a similar way to the P3 amplitudes (with window of 378–418 ms). Data from the Ignore condition were available for all 14 subjects. One subject had to terminate her participation due to illness half way through the experiment, so only 13 subjects were available for the Attending condition.

## Results

### Questionnaire Data on Self-Reported Alert Familiarity, Device Ownership and Use

A *t*-test analysis of the sound familiarity ratings did not significantly differ between the Outlook alert or the Android alert. The average familiarity rating across both alerts was 2.88 on a scale of 1–5 (with 5 being extremely familiar, 1 being not at all familiar). In terms of primary alert usage, about 31% of participants reported that the Outlook alert was their primary alert and 21% reported Android as their primary alert.

### Behavioral Data

The behavioral data of participants were analyzed to look at effects on reaction time, hit rate and *d*′ measures for the target (alert) sounds. It was found that *d*′ measures (with targets measured against their distractors) significantly differed for the two target type sounds (*F*_1,27_ = 8.062, *p* < 0.01), with the perceptual sensitivity greater for the Android alert than for the Outlook alert (mean = 4.31 vs. mean = 3.42, respectively). Similarly, the Outlook targets also had smaller *d*′ (mean = 4.39) against the standard noise bursts compared to the Android targets (mean = 5.51), *F*_1,27_ = 7.65, *p* < 0.05. The hit rate and reaction time to Outlook and Android alert sounds did not significantly differ and did not differ as a function of whether the participant reported that they had that sound as a primary alert on their own devices. The dissociation between HR/RT and *d*′ measures confirmed our third hypothesis that the physical discrimination between stimuli did not necessarily affect the RT or hit rate negatively.

### ERP Data

The ERP data was analyzed for three components in the sample. The first was the mismatch negativity component, which was analyzed as an index of pre-attentive processing and change detection. The second component was the P3 component, which we expected as either attentional switch in ignore conditions (P3a) or direct target detection (P3b) in attend conditions. In addition, a third component (late positive component, which we acknowledge could simply be a late P3b) was seen in the frontal regions, which was of interest, and this was also made subject to statistical analysis.

#### MMN Component

**FIGURE 1:**
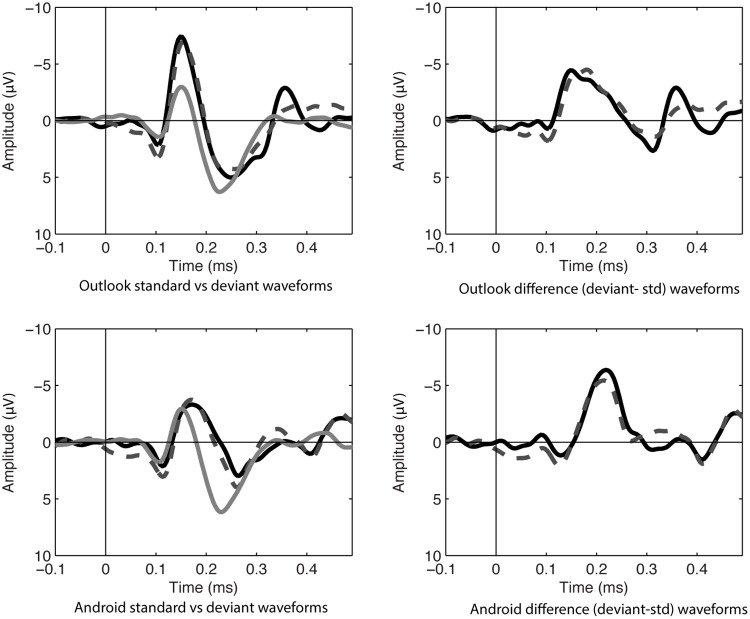
ERP waveforms in the ignore condition at Fz of standard (noise) and deviant sounds on left hand panels (Target Alert = black, Matched Distractor deviant = dashed gray, white noise standards = gray). Difference waves on right hand panels (Alert deviant-standard noise = black, Matched distractor-standard noise = dashed gray).

**FIGURE 2:**
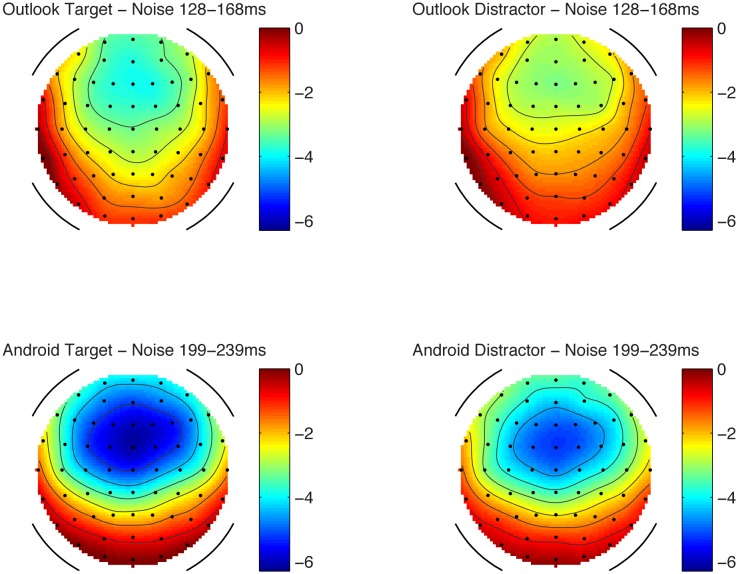
Scalp topography maps for the MMN component in the ignore condition. Scale in microvolts (μV).

In the ignore condition, there was a significant mismatch negativity at frontal and central sites, when tested with one-sampled *t*-test. The largest value appeared to be at Fz, consistent with the MMN morphology, see **Table 1** and **Figures 1, 2**.

**Table 1:** Mean and SD MMN amplitudes at Fz in ignore condition, along with *t*-test values against zero.

Sound type	Mean amplitude (μV) (*SD* in brackets)	*t*-value (df = 13)	*p*-value
Outlook target	-3.87 (2.34)	-6.18	<0.01
Outlook distractor	-3.213 (2.74)	-4.39	<0.01
Android target	-5.96 (2.55)	-8.73	<0.01
Android distractor	-5.03 (2.51)	-7.30	<0.01


Using a repeated measures ANOVA at Fz comparing alert type (Outlook vs. Android) and target status (targets vs. distractors) in the ignore condition, it was found that matched distractors had lower MMN amplitudes compared to the targets (*F*_1,13_ = 6.945, *p* < 0.05) and Android alerts had larger MMNs compared to Outlook alerts (*F*_1,13_ = 7.793, *p* < 0.05). No interactions were significant. Lateral electrodes (F3 and F4) were also analyzed for laterality effects but this was not statistically significant.

**FIGURE 3:**
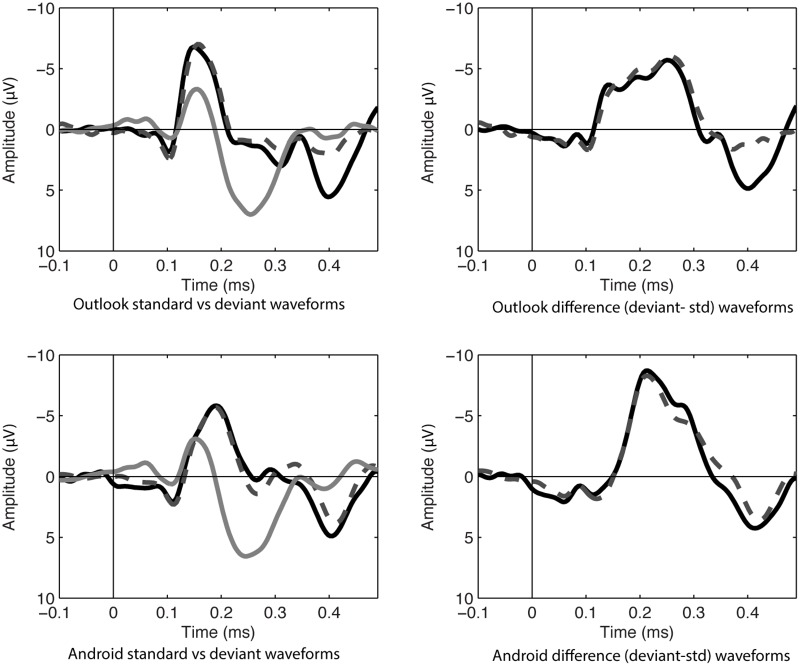
ERP waveforms in attending condition at Fz of standard (noise) and deviant sounds on left hand panels (Target Alert = black, Matched Distractor deviant = dashed gray, white noise standards = gray). Difference waves on right hand panels (Alert deviant-standard noise = black, Matched distractor-standard noise = dashed gray).

**FIGURE 4:**
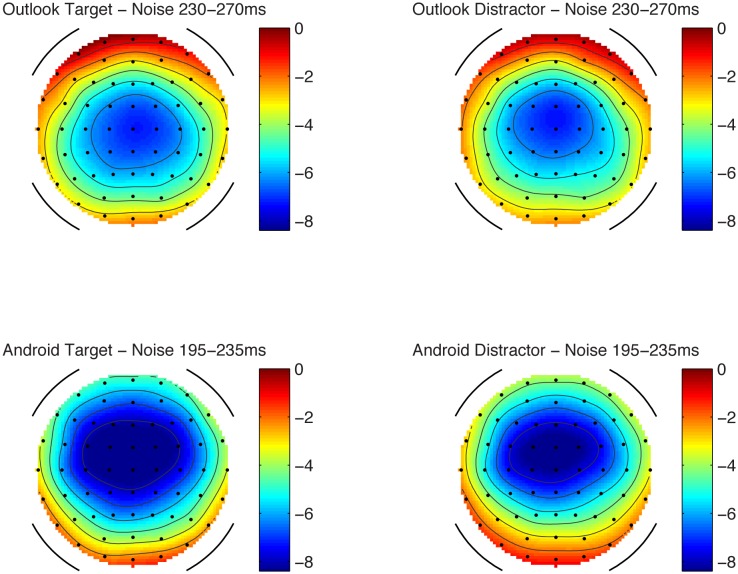
Scalp topography maps for the MMN component in the attending condition. Scale in microvolts (μV).

In the attending condition, MMN amplitudes were also significant against a zero value and maximal at Fz, see values in **Table 2** and **Figures 3, 4**.

**Table 2:** Mean and SD MMN amplitudes at Fz in attending condition, along with *t*-test values against zero.

Sound type	Mean amplitude (μV) (*SD* in brackets)	*t*-value (df = 12)	*p*-value
Outlook target	-5.41 (4.97)	-3.52	<0.01
Outlook distractor	-5.76 (4.81)	-4.32	<0.01
Android target	-8.23 (3.53)	-8.41	<0.01
Android distractor	-7.82 (3.80)	-7.42	<0.01


Using a repeated measures ANOVA at Fz comparing alert type (Outlook vs. Android) and target status (targets vs. distractors) in the attending condition, Android alerts had larger MMNs compared to Outlook alerts (*F*_1,12_ = 4.93, *p* < 0.05), but there were no significant differences between targets and distractors. No interactions were significant. Lateral electrodes (F3 and F4) were also separately analyzed for laterality but this was not statistically significant, however, there was a statistically significant difference between frontal and fronto-central and central electrodes, with Fz having a larger maximum amplitude compared with FCz or Cz (*F*_1,12_ = 42.05, *p* < 0.01), which is consistent with MMN morphology. Although the figures reported here included Fz in the main analysis (as this was the most reliable when *t*-tests against zero were run), further re-analysis of all sites included in the ANOVA did not change the result.

Mismatch negativity amplitudes between attending and ignore condition were also compared. It was found that MMN amplitudes were larger in the attend condition compared to the ignore condition (*F*_1,12_ = 42.05, *p* < 0.05). The differences between ignore and attend conditions are explained by established effects of N2b enhancement (Näätänen et al., 2007). In terms of latency, MMN peaked earlier within the ignore condition for the Outlook alerts (mean = 165 ms) compared to the Android alerts (mean = 218 ms), *F*_1,13_ = 96.128, *p* < 0.01. By contrast, in the attending condition, the Outlook alerts peaked later (mean = 255 ms) compared to the Android alerts (mean = 219 ms), *F*_1,12_ = 38.328, *p* < 0.01. No other main effects or interactions were significant in the MMN latency. Taken together, there appears to be some discrepancy between latency data such that MMN was unusually earlier for smaller waves for the Outlook alerts in the ignore condition, whereas the reverse (and more common pattern) was true for the attending condition, with larger waves in the Android alerts showing earlier MMN peaks.

#### P3a Component

**FIGURE 5:**
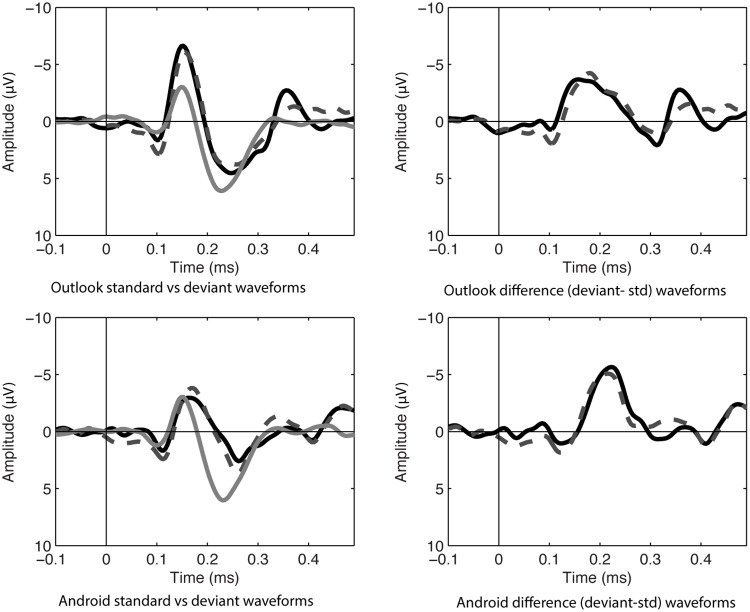
ERP waveforms in ignore condition at Cz of standard (noise) and deviant sounds on left hand panels (Target Alert = black, Matched Distractor deviant = dashed gray, white noise standards = gray). Difference waves on right hand panels (Alert deviant-standard noise = black, Matched distractor-standard noise = dashed gray).

**FIGURE 6:**
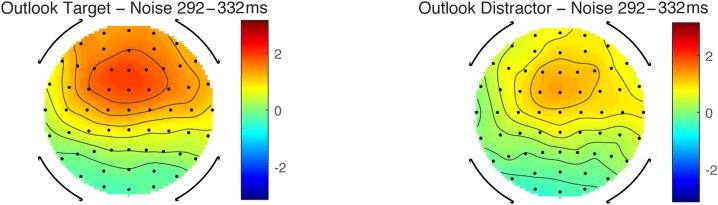
Scalp topography maps for the P3 component in the ignore condition. Scale in microvolts (μV).

In the ignore condition, there was a significant P3a at central sites, when tested with one-sampled *t*-test, but only for the Outlook and matched Outlook distractor. The largest value appeared to be at Cz, consistent with the P3 morphology, see **Table 3** and **Figures 5, 6**.

**Table 3:** Mean and SD P3a amplitudes at Cz in ignore condition for Outlook sounds and matched distractors, along with *t*-test values against zero.

Sound type	Mean amplitude (μV) (*SD* in brackets)	*t*-value (df = 13)	*p*-value
Outlook target	1.44 (1.82)	2.95	=0.01
Outlook distractor	0.97 (1.56)	2.31	<0.05


*Android sounds did not elicit significant positive amplitudes within the expected P3a latency range.*

Analysis of P3a amplitudes only included Cz and C4 electrodes in the Outlook and Outlook distractor conditions as these were the only significant signals. There was no significant difference between target and distractor sounds and nor was there a significant effect of site.

In terms of P3a latency within the ignore condition, therignore condition, there was no statistical difference between latency to targets and distractors. No other main effects or interactions were significant. Together, these results lend support for our second hypothesis (H2) that P3a would differ as a function of the meaningfulness of the stimuli. It was interesting that despite the smaller d’ measure of the Outlook tone, that this still resulted in a larger P3a wave for the target compared to the distractor. This is likely to reflect attention directed toward the meaning of the stimuli.

For the attending condition, there was no positive signal that achieved statistical significance in the expected latency range (230–370 ms) and at the expected sites of Cz in any of the conditions. Instead a later positivity (around 400 ms) was seen and is analyzed and discussed in the subsequent sections.

#### Frontal Late Positive Component

In the attend condition, there was a significant late frontal positive component at frontal and central sites, when tested with one-sampled *t*-test. The largest value appeared to be at Fz, see **Table 4** and **Figures 1** (left hand panel of MMN plots), 7.

**Table 4:** Mean and SD late frontal positive component amplitudes at Fz in ignore condition, along with *t*-test values against zero.

Sound type	Mean amplitude (μV) (SD in brackets)	*t*-value (df = 12)	*p*-value
Outlook target	4.42 (3.59)	4.44	<0.01
Outlook distractor	1.30 (3.17)	1.48	>0.05
Android target	3.55 (2.59)	4.94	<0.01
Android distractor	2.19 (2.40)	3.29	<0.01


*Although the Outlook distractor positive amplitude was not statistically significantly different from zero, it is included in this table for comparison, but was significant at other sites (FC and C electrodes).*

**FIGURE 7:**
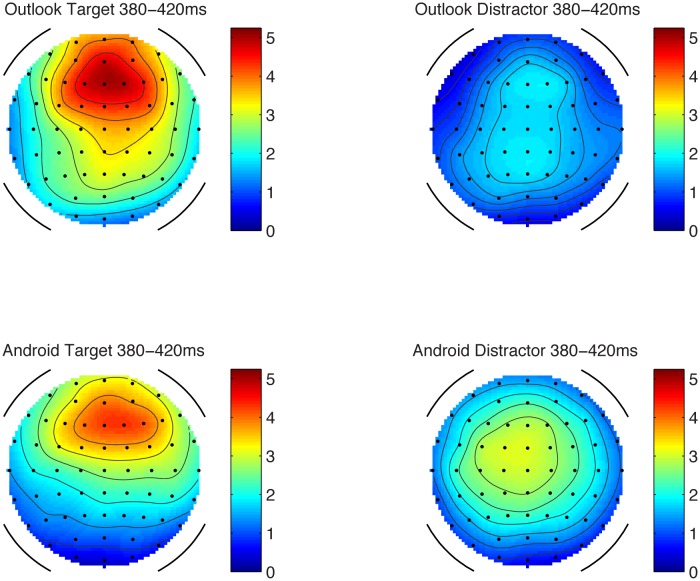
Scalp topography maps for the late positive component in the attending condition. Scale in microvolts (μV).

The amplitudes appeared largest at Fz compared to Cz. However, there was no statistically significant difference between FCz and Fz. Nonetheless, Fz was chosen for analysis as it did have the largest signal. Analysis of laterality (including F3 and F4 sites) did not show any statistically significant differences between right and left hand sites.

Analysis at Fz of stimulus type and target status showed that the target alert sounds generated larger frontal positive component amplitudes compared to the matched distractor sounds, (*F*_1,12_ = 16.47, *p* < 0.05), but Outlook vs. Android ‘types’ of sounds did not significantly differ in amplitude. There was a significant interaction between target status and alert type, such that the difference in late positive component amplitude was larger for the Outlook target tone than its matched distractor, whereas for the Android alert, the late positive component amplitude was more similar compared to its matched distractor (*F*_1,13_ = 5.307, *p* < 0.05). The Outlook target tone appeared to have the largest amplitude out of all the sounds. Interestingly, in terms of latency, these analyses only showed that the frontal positivity was significantly earlier (398 ms vs. 408 ms) for the Outlook sounds compared to the Android sounds (*F*_1,13_ = 8.246, *p* < 0.05). However, there was no significant effect of target status nor interaction between alert type and target status.

In summary, the data suggest that there is a response to the status of these sounds as ‘targets’ as indexed by this late positive component. Moreover, the amplitude of the wave does not appear to be the result of purely physical discriminability, as evidenced by *d*′, since the Outlook tone appeared to have the largest amplitude (and yet the smallest *d*′).

#### Correlations Between Questionnaire and Behavioral Data

As we were interested in whether there were correlations between high tendencies of problematic work-patterns/job involvement and rumination (as indexed by our questionnaires) and behavioral target detection performance, we correlated these measures.

In correlations of questionnaire measures and target detection performance (hit rate, *d*′ and RT), only the Outlook alert reaction time correlated (negatively) with the index of job involvement (*r* = -0.482, *p* < 0.01, which is significant when using Bonferroni correction). In this sense, there was evidence of partial support for our hypothesis that there would be a correlation between high tendencies of problematic work-patterns/job involvement and rumination (as indexed by our questionnaires) and behavioral target detection performance. Together with the finding that later components of ‘attentional switch’ (indexed by P3a) and conscious attention (indexed by late positive wave around 400 ms) effects were strongest for the Outlook sounds, this may suggest that the Outlook sounds symbolize a ‘work context’ more than the Android alert. No other measures of target detection nor the ERP components were correlated with the questionnaire measures when controlling for family wise error rates.

## Discussion

This study broadly sought to investigate the pre-attentive processing and potential attentional switches to new communication technology alerts, in order to determine whether there are enhanced responses to these stimuli in comparison to matched stimulus controls. In parallel, we also sought to determine whether cognitive processes underlying detection of these stimuli were correlated with measures of poorer work-life balance.

Our first hypothesis (H1) examined whether the effects of pre-attentive processing (as indexed by MMN) of physical difference and meaning were dissociated. In this respect, the results are mixed for the MMN data. In the ignore condition, target status did have an effect (consistent with the data on MMN to meaningful stimuli or categories), but this effect did disappear with conscious, directed attention. On the other hand, the Android alerts, did, in general have larger amplitudes compared to the Outlook tones, which is likely to be reflected in the fact that the Android alerts were more perceptually discriminable (as indexed by *d*′) than the Outlook alerts. This latter finding is explained by the existing body of evidence suggesting that MMN increases with increasing physical difference (e.g., Jaramillo et al., 2000). The MMN was also enhanced with attention, which is likely to be due to the elicitation of an N2b (Näätänen et al., 2007).

With respect to the second hypothesis, we sought to examine whether ERP indices of attentional processing – e.g., P3a or possibly later positive components as found by Roye et al. (2007) would differ between the NCT alerts and matched stimulus controls. Within the ignore condition, there was only a significant P3a for the Outlook sounds, not the Android Targets nor distractors, despite Android sounds eliciting larger MMNs. In the attend condition, there was no clear P3a, but instead a later positivity (around 400 ms) that was maximal at frontal sites. Analyses of this component showed that there were differences between alerts and matched distractors (such that the true alerts had higher amplitudes). However, there was an interaction between target status and sound type, such that the positivity was greatest for the Outlook sound and the difference far more pronounced between it and its matched distractor compared to the Android group of sounds. It was curious that this positive component was frontally distributed rather than centrally or even parietally as one might typically expect of P3-type responses. However, looking at other studies that have used ‘meaningful sounds,’ it appears that frontal distributions in positive waves can be expected. For example, Uther et al. (2006) study of morse code stimuli showed a frontal distribution, as well as ((s))Tervaniemi et al. (2016) use of musical stimuli. Roye et al. (2007) also identified a positive wave that was more frontal in their studies of messaging alerts too. Further research in this area would benefit from a more detailed exploration of whether meaningful stimuli in general (not just messaging alerts) elicit late positivities that are differently (i.e., frontally) distributed and source localized differently to ‘regular’ late positivities such as P3 responses. The ultimate question of whether this is a separate component remains to be empirically tested, but given the unusual scalp topography, we have tentatively distinguished this from the ‘regular’ P3 responses.

With respect to the hypothesis that there would be a correlation between high tendencies of problematic work-patterns/job involvement and rumination (as indexed by our questionnaires) and behavioral target detection performance, it appears that there was only a correlation between faster reaction times to Outlook sounds with higher job involvement. Interestingly, this finding did not appear to be due to increased physical discrimination of that sound as *d*′ measures were actually smaller for the Outlook alert than the Android sound. Data on usage and familiarity of both sounds did not indicate any difference between either the Outlook or Android sound alerts. A likely explanation of these effects would be that the *meaning* (significance) of that sound is signifying a readiness to respond. When comparing the everyday usage of the two sound types, Outlook was an email sound, whereas the Android sound was a text message alert. One thing that could be tested in the future is whether it is the type of message (email vs. text message) or context (work or non-work) that might be contributing to this – although the two effects of work context and message type (email vs. text) is always invariably likely to be confounded. It is most likely that the meaning attached the Outlook sound signals work-related activities for the user. Hence, a correlation with job involvement makes sense. Nonetheless, further work with more fine-grained controls is needed to test this.

Finally, with respect to our final hypothesis, we saw a dissociation between RT and d’ responses, such that target/meaningful stimuli may have quicker reaction times despite a smaller *d*′. Hence, it does appear despite both messaging alerts being responded to equally quickly by participants, they were not equal in terms of their physical discriminability (as indexed by *d*′, which takes into account error rates). In other words, although participants were responding to the Outlook stimuli as quickly as they were for Android stimuli, they found it harder to distinguish between the target and distractors. This matters in terms of the interpretation of the ERP effects. It means that any enhancements we see with respect to ERP components for Outlook stimuli are not likely to be due simply to physical discriminability, but instead meaning or significance of the stimuli. Further work would of course be needed to further establish whether similar patterns hold for other messaging alert stimuli or any kind of meaningful stimuli (e.g., musical stimuli).

In summary, the data presented here was an initial exploration of the neural and cognitive processing of common messaging alerts and the potential link between heightened sensitivity and work-life balance measures. Of course, it should be noted that the sample of participants might have been too small to see modest effects of a relationship between ERP measures and work-life balance. Future work with larger ERP datasets would address this and will be the focus of future research. Similarly, more controlled testing of messaging alerts that are definitively linked to different work contexts (e.g., home vs. work) would also be helpful. Finally, source localisation of the late frontal positivity found in this study and previous studies on meaningful stimuli would serve to determine whether there are specific neural mechanisms that the brain uses to tune to meaningful and significant stimuli as compared to other sounds in our environment.

## Conclusion

Together these data suggest that common messaging alerts do elicit brain responses that might be unique to sounds that have meaning and significance (albeit at a later stage of processing than we initially predicted). Nonetheless, further follow up work with different alerts to those used here that are coupled with a more detailed assessment of their perceived associations (i.e., whether they associated certain sounds with a work context compared to others) is required. We also had a relatively modest sample size for both ERP and behavioral datasets, so it would be prudent to replicate these effects with much larger sample sizes. Finally, it would also be useful to include autonomic arousal measures (i.e., skin conductance, heart rate variability) to determine whether the brain measures correlate with autonomic indices of stress and arousal.

## Ethics Statement

The study was carried out in accordance with the BPS guidelines for conducting research with human participants, with written consent from all subjects. All subjects gave written informed consent in accordance with the Declaration of Helsinki. The protocol was approved by the University of Winchester Research and Knowledge Exchange Ethics Committee.

## Author Contributions

MU conceived the research topic, devised experimental protocol, conducted the statistical analysis, and led in write-up. RJ contributed to the experimental design, contributed to the final write-up conducted ERP data collection, and analysis of ERP data/figures. MC advised on the experimental design (especially questionnaire measures), organized participant recruitment and relevant ethical approval. MC also contributed to the literature review regarding work-life balance and assisted with the write-up and editing.

## Conflict of Interest Statement

The authors declare that the research was conducted in the absence of any commercial or financial relationships that could be construed as a potential conflict of interest.
